# TRPC3 Regulates the Proliferation and Apoptosis Resistance of Triple Negative Breast Cancer Cells through the TRPC3/RASA4/MAPK Pathway

**DOI:** 10.3390/cancers11040558

**Published:** 2019-04-18

**Authors:** Yan Wang, Yan-Xiang Qi, Zenghua Qi, Suk-Ying Tsang

**Affiliations:** 1 School of Life Sciences, The Chinese University of Hong Kong, Hong Kong, China; 1155070144@link.cuhk.edu.hk (Y.W.); YanxiangQi@link.cuhk.edu.hk (Y.-X.Q.); zenghuaqi@gmail.com (Z.Q.); 2 State Key Laboratory of Agrobiotechnology, The Chinese University of Hong Kong, Hong Kong, China; 3 Key Laboratory for Regenerative Medicine, Ministry of Education, The Chinese University of Hong Kong, Hong Kong, China; 4 Centre for Novel Biomaterials, The Chinese University of Hong Kong, Hong Kong, China

**Keywords:** TRPC3, calcium influx, triple-negative breast cancer, apoptosis resistance, RASA4, MAPK pathway

## Abstract

Currently, there is no effective molecular-based therapy for triple-negative breast cancer (TNBC). Canonical transient receptor potential isoform 3 (TRPC3) was previously shown to be upregulated in breast cancer biopsy tissues when compared to normal breast tissues. However, the biological role of TRPC3 in breast cancer still remains to be elucidated. In this study, subcellular fractionation followed by Western blot and immunocytochemistry showed that TRPC3 was over-expressed on the plasma membrane of TNBC line MDA-MB-231 when compared to an estrogen receptor-positive cell line MCF-7. TRPC3 blocker Pyr3 and dominant negative of TRPC3 attenuated proliferation, induced apoptosis and sensitized cell death to chemotherapeutic agents in MDA-MB-231 as measured by proliferation assays. Interestingly, Ras GTPase-activating protein 4 (RASA4), a Ca^2+^-promoted Ras-MAPK pathway suppressor, was found to be located on the plasma membrane of MDA-MB-231. Blocking TRPC3 decreased the amount of RASA4 located on the plasma membrane, with concomitant activation of MAPK pathways. Our results suggest that, in TNBC MDA-MB-231 cells, Ca^2+^ influx through TRPC3 channel sustains the presence of RASA4 on the plasma membrane where it inhibits the Ras-MAPK pathway, leading to proliferation and apoptosis resistance. Our study reveals the novel TRPC3-RASA4-MAPK signaling cascade in TNBC cells and suggests that TRPC3 may be exploited as a potential therapeutic target for TNBC.

## 1. Introduction

Breast cancer is one of the leading heterogeneous diseases in women worldwide which can be divided into several subtypes [1,2]. According to the statistics from the National Cancer Institute (SEER 18, 2008–2014), the 5-year relative survival rate of female patients with localized breast cancer is 98.7%, whereas the rate for the female patients with metastatic breast cancer is only about 27.0%. Surgery in combination with endocrine therapy can provide better treatments for the patients with estrogen receptor (ER) positive, progesterone receptor (PR) positive and human epidermal growth factor receptor 2 (HER2) positive breast cancer [3]. The treatment of triple-negative breast cancer (TNBC), a highly metastatic subtype, still remains challenging due to the lack of targeted therapy.

Apoptosis is a key regulator of tissue homeostasis [4]. An imbalance between cell proliferation and apoptosis promotes tumorigenesis. Chemotherapy, radiation therapy and immunotherapy, through inducing DNA damage and triggering apoptosis of cancer cells, are major treatment strategies for TNBC [5,6]. However, the side effects of these conventional treatments are severe. Antibody-drug conjugates (ADCs), which can allow exact targeting to tumour cell-surface proteins, are a new class of therapeutic agents for targeted cancer therapy [7]. Therefore, identification of differentially expressed cell-surface proteins in TNBC is deemed necessary for an effective and specific treatment.

Transient receptor potential (TRP) channels, a group of non-selective cation channels, modulates a diversity of cellular physiological traits. Differential expression as well as dysregulation of specific TRP channels have presented positive correlations with different breast cancer subtypes. Upregulated TRP channels worsen breast cancer progression through increasing cell proliferation, migration and invasion. Thus, TRP channels have been proposed as potential breast cancer diagnostic markers and therapeutic targets [8,9,10].

Canonical TRP isoform 3 (TRPC3) channel was reported to be upregulated in breast cancer biopsy tissues when compared to normal breast tissues [11]. However, the biological role of TRPC3 in breast cancer still remains to be elucidated. In the present study, we aimed to investigate if TRPC3 is responsible for the proliferation and apoptosis resistance of the TNBC cells, and, if yes, the underlying mechanisms involved.

## 2. Results

### 2.1. Upregulation of TRPC3 on the Plasma Membrane of Triple-Negative Breast Cancer (TNBC) Cells MDA-MB-231

**Figure 1:**
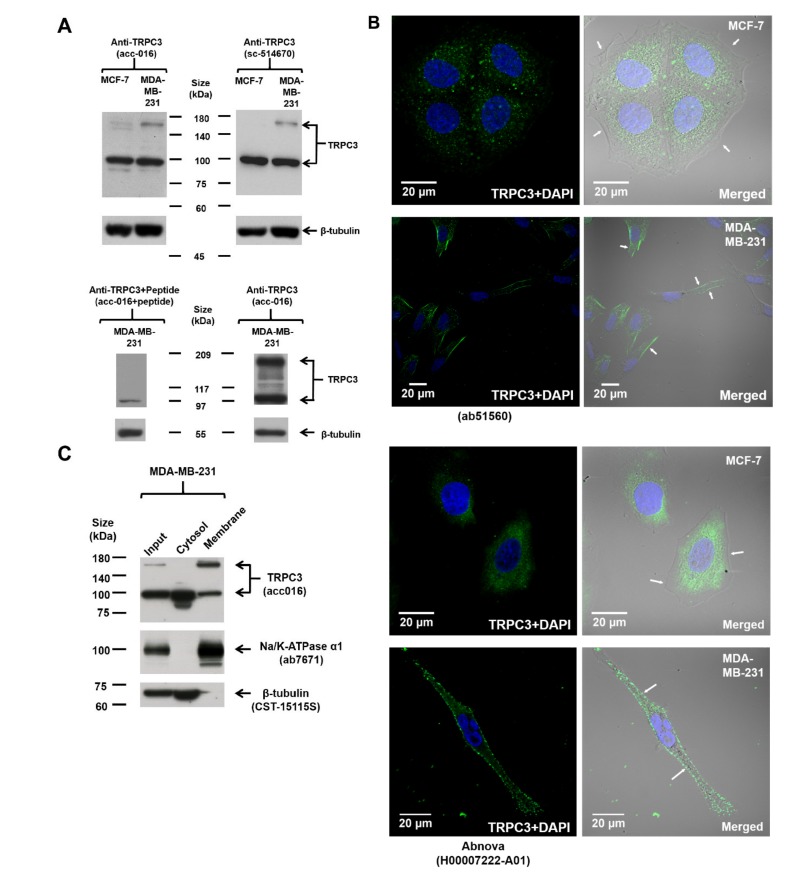
TRPC3 was over-expressed on the plasma membrane of MDA-MB-231. (**A**) representative Western blots showing the expression of TRPC3 in MCF-7 and MDA-MB-231. TRPC3 protein (~100 kDa) was expressed in both MCF-7 and MDA-MB-231, whereas TRPC3 protein represented by the band between 140 and 180 kDa was over-expressed in MDA-MB-231. Membranes were incubated with two different TRPC3 antibodies (Alomone Labs, Jerusalem, Israel and Santa Cruz, Dallas, TX, USA) and consistent expression patterns were detected. β-tubulin was used as an internal control. Corresponding bands became faded or disappeared when the membrane was incubated with TRPC3 antibody pre-incubated with its corresponding peptide antigen (Alomone Labs), suggesting the specificity of the bands. (**B**) representative confocal images showing the subcellular localization of TRPC3 (green) in MCF-7 and MDA-MB-231. Cells were incubated with two different TRPC3 antibodies (Abcam, Cambridge, UK and Abnova, Taipei, Taiwan). Nuclei were stained with DAPI (blue). Merging fluorescence images with bright field images revealed that TRPC3 was over-expressed on the plasma membrane of MDA-MB-231 when compared to MCF-7. Plasma membrane positions were indicated by white arrows. Scale bar: 20 μm. (**C**) subcellular fractionation followed by Western blot analysis confirmed that the over-expressed TRPC3 protein represented by the band between 140 and 180 kDa was enriched in the membrane fraction of MDA-MB-231. Na/K-ATPase α1 was used as a membrane protein marker and β-tubulin was used as a cytosolic protein marker.

The expression of TRPC3 in MCF-7 and MDA-MB-231 was examined by Western blot. Immunoblots conducted using two different TRPC3 antibodies revealed consistent TRPC3 expression patterns. Two discrete bands, one at around 100 kDa and one located between 140 and 180 kDa, were detected (Figure 1A; Figure S1A), similar to the reported sizes of TRPC3 in human ovarian cancer cell line SKOV3 [12]. The intensity of both bands was greatly diminished if the anti-TRPC3 was pre-incubated with its antigenic peptide (Figure 1A), suggesting that both bands are specific bands. The band at around 100 kDa which matched the expected size of human TRPC3 protein was detected in both MCF-7 and MDA-MB-231, whereas the band between 140 and 180 kDa was much stronger in MDA-MB-231 (Figure 1A; Figure S1A). Interestingly, this upregulated band between 140 and 180 kDa was found to be DTT-sensitive (Figure S1B) and is speculated to represent a dimeric TRPC3 band [13,14,15].

To pinpoint the sub-cellular localization of TRPC3 in MCF-7 and MDA-MB-231, immunocytochemistry was performed followed by confocal fluorescence microscopy. Cells were stained with two different TRPC3 antibodies. TRPC3 was found to be over-expressed on the plasma membrane of MDA-MB-231 when compared to MCF-7 (Figure 1B). To further confirm the expression of TRPC3 in MDA-MB-231, subcellular fractionation followed by Western blot analysis was performed. The upregulated band between 140 and 180 kDa was only present in the membrane fraction but not the cytosolic fraction of MDA-MB-231 (Figure 1C). In addition, this band between 140 and 180 kDa was not detected in the membrane fraction of MCF-7 (Figure S1A). All of these data suggested that TRPC3 was over-expressed on the plasma membrane of MDA-MB-231.

### 2.2. TRPC3 Regulated Calcium Influx, Cell Proliferation and Apoptosis of MDA-MB-231

**Figure 2:**
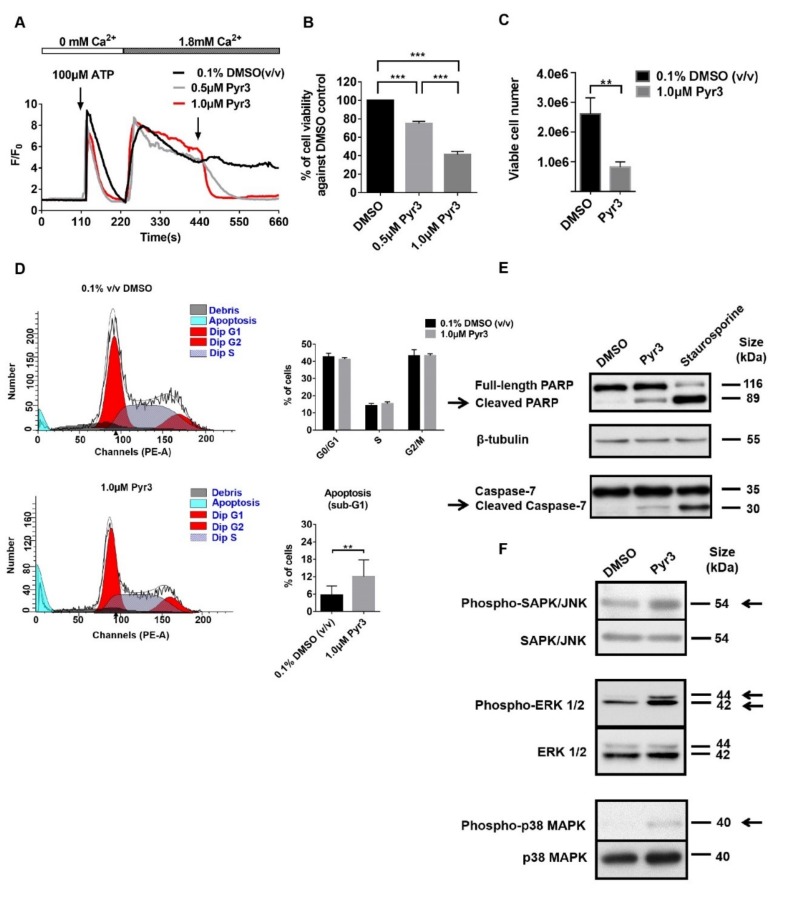
TRPC3 regulated calcium influx, proliferation and apoptosis of MDA-MB-231. (**A**) representative Ca^2+^ imaging traces reflected changes in the level of cytosolic free calcium over time in MDA-MB-231. Average fluo-4 fluorescence intensity was transiently increased in response to 100 μM ATP when external Ca^2+^ was absent. Addition of external calcium (1.8 mM) led to an increase in fluorescence intensity; a marked decrease of the fluorescence intensity was observed when 0.5/1.0 μM Pyr3 was applied. Our results showed that TRPC3 blocker Pyr3 abolished ATP-induced Ca^2+^ influx in MDA-MB-231. F/F0: fluorescence (F) normalized to baseline fluorescence (F0). Traces of fluorescence intensity are average of at least three independent experiments, with 75–100 cells measured in total; (**B**) blocking TRPC3 by Pyr3 (0.5/1.0 μM for 72 h) decreased the percentage of viable MDA-MB-231 cells in a concentration-dependent manner when compared to DMSO control as measured by an MTT assay. OD570 values of 0.1% DMSO (*v*/*v*) solvent control group was set as 100% of cell viability. Values are mean ± SEM (*n* = 5). *** *p* < 0.001; (**C**) blocking TRPC3 by Pyr3 (1.0 μM for 120 h) attenuated the proliferation of MDA-MB-231 as measured by trypan blue exclusion assay. Initial seeding number of MDA-MB-231 cells was 2 × 10^5^ and viable cells were counted after 5-day DMSO/ Pyr3 treatment. Values are mean ± SEM (*n* = 3). ** *p* < 0.01; (**D**) blocking TRPC3 by Pyr3 (1.0 μM for 120 h) increased DNA damage with accumulation of cells in the sub-G1 phase but did not affect cell cycle distribution of viable cells as measured by cell cycle analysis. Values are mean ± SEM (*n* = 3). ** *p* < 0.01; (**E**) representative Western blots showing that levels of cleaved caspase-7 and cleaved PARP were increased in Pyr3-treated MDA-MB-231 cells when compared to DMSO control group. MDA-MB-231 cells treated with 0.1 μM staurosporine (apoptosis inducer) for 24 h was used as positive control for detection of bands of cleaved caspase-7 and PARP proteins. β-tubulin was used as an internal control. Results showed that blocking TRPC3 by Pyr3 (1.0 μM for 72 h) induced apoptosis of MDA-MB-231 in a caspase-dependent manner; (**F**) representative Western blots showing that levels of phosphorylated p38 MAPK, ERK1/2 and JNK were all increased in Pyr3-treated MDA-MB-231 cells. Total p38 MAPK, ERK1/2 and JNK were also detected. Results showed that blocking TRPC3 by Pyr3 (1.0 μM for 72 h) activated MAPK pathways in MDA-MB-231 cells.

Functional presence of TRPC3 in MDA-MB-231 cells was measured by Ca^2+^ imaging assay. In the presence of external solution containing 1.8 mM free calcium, Pyr3, a specific TRPC3 blocker [16], abolished ATP-induced Ca^2+^ influx in MDA-MB-231 (Figure 2A). The result suggested that TRPC3 was functionally present in MDA-MB-231. In addition, MTT assay showed that Pyr3 decreased the percentage of viable MDA-MB-231 in a concentration-dependent manner when compared to the solvent control group (Figure 2B). Consistently, with an initial seeding number of 2 × 10^5^ cells and 5-day treatment of Pyr3 or solvent, cell counting by trypan blue exclusion assay revealed that Pyr3 decreased the number of viable MDA-MB-231 when compared to the solvent control group (Figure 2C). To identify the underlying causes of the Pyr3 effect, cell cycle analyses were performed. Pyr3 (1.0 μM for 120 h) caused an increase in the percentage of MDA-MB-231 accumulated in the sub-G1 phase but did not affect cell cycle distribution of viable cells (Figure 2D). Typical apoptotic morphological changes, including cell shrinkage, membrane blebbing, mitochondrial fragmentation and nuclear condensation, were observed in MDA-MB-231 cells after 1.0 μM Pyr3 treatment for 8 h (Figure S2A). Cell shrinkage and nuclear condensation were also observed in Ad-DN-TRPC3-infected MDA-MB-231 cells (Figure S2B).

Our results suggested that blocking TRPC3 induced apoptosis with increasing DNA damage. Levels of caspase-3/7 and cleaved caspase-3/7, poly (ADP-ribose) polymerase (PARP) and cleaved PARP, phosphorylated and total p38 MAPK, ERK1/2 and JNK proteins were examined by Western blot. Pyr3 caused an upregulation of cleaved caspase-3/7 and cleaved PARP (Figure 2E; Figure S3), suggesting that blocking TRPC3 would increase DNA damage and induce apoptosis in a caspase-dependent manner. Interestingly, levels of phosphorylated p38 MAPK, ERK1/2 and JNK proteins were all increased upon Pyr3 treatment (Figure 2F), indicating that blocking TRPC3 would activate MAPK pathways. Moreover, among all the MAPK subfamilies, the level of phosphorylated ERK1/2 was markedly increased in Pyr3-treated cells. All of these data suggested that TRPC3 positively contributes to the proliferation of MDA-MB-231 and acts as an anti-apoptotic regulator.

### 2.3. Dominant Negative (DN) of TRPC3 Attenuated Cell Proliferation, Induced Cell Apoptosis and Sensitized Cell Death to Chemotherapeutic Agents in MDA-MB-231

**Figure 3:**
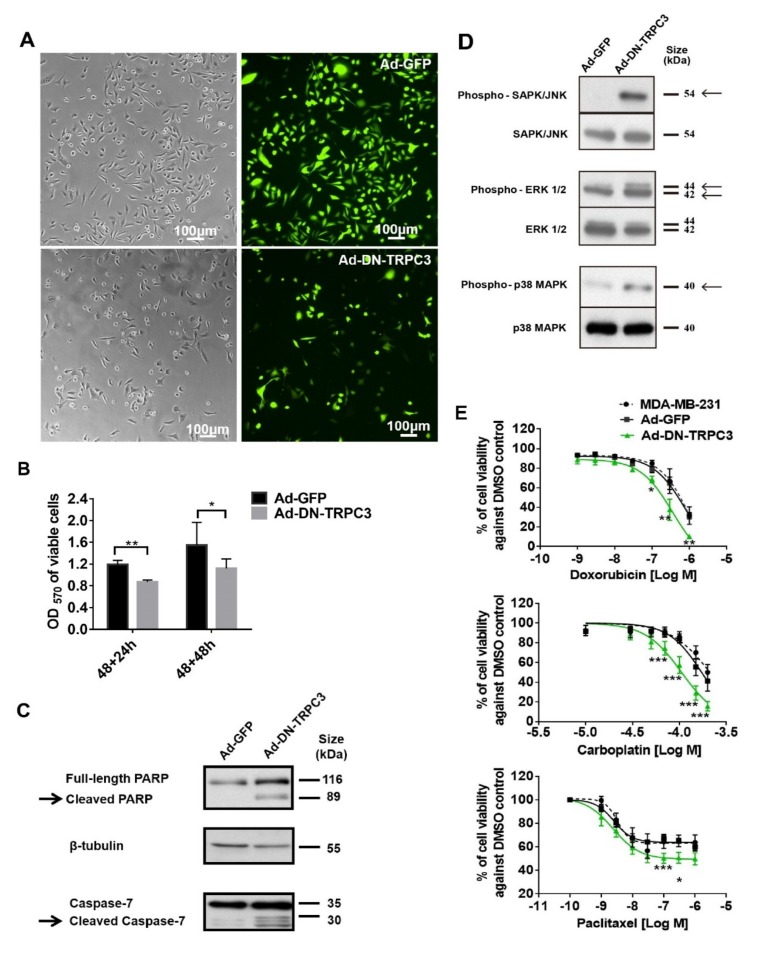
Dominant negative (DN) of TRPC3 attenuated proliferation, induced apoptosis and sensitized cell death to chemotherapeutic agents in MDA-MB-231. (**A**) recombinant adenoviruses (Ad) harboring GFP (Ad-GFP) or DN of TRPC3 (Ad-DN-TRPC3) were used to infect MDA-MB-231 for 48 h. Infection efficiency was determined by the percentage of cells with GFP fluorescence and was typically assessed to be 90–95%; (**B**) DN of TRPC3 attenuated cell proliferation as measured by MTT assay performed at 24 and 48 h after adenoviruses withdrawal. OD570 values of viable cells were compared between Ad-GFP and Ad-DN-TRPC3-infected group at different time points. Values are mean ± SEM (*n* = 3). * *p <* 0.05, ** *p* < 0.01; (**C**,**D**) representative Western blots showing that DN of TRPC3 (**C**) induced apoptosis in a caspase-dependent manner and (**D**) activated MAPK pathways in MDA-MB-231 cells. Similar results were obtained when the cells were incubated with Pyr3 (cf. Figure 2); (**E**) DN of TRPC3 sensitized cell death to chemotherapeutic agents in a concentration-dependent manner as measured by MTT assay. Ad-GFP-infected cells and non-stimulated MDA-MB-231 cells presented similar trends of decrease in cell viability in response to doxorubicin, carboplatin or paclitaxel. Values are mean ± SEM (*n* = 3). * *p <* 0.05, ** *p* < 0.01 and *** *p* < 0.001 versus Ad-GFP control.

To further study the effect of functional knockdown of TRPC3, recombinant adenoviruses harboring of GFP and DN of TRPC3 [17] were used to infect MDA-MB-231 cells. Consistent with the effect of TRPC3 blocker Pyr3, DN of TRPC3 attenuated cell proliferation and induced apoptosis via activating MAPK pathways in MDA-MB-231 (Figure 3A–D). In addition, Ad-DN-TRPC3-infected MDA-MB-231 were more sensitive to apoptotic cell death caused by chemotherapeutic agents (doxorubicin, carboplatin and paclitaxel) as measured by MTT assay (Figure 3E).

### 2.4. TRPC3 Blockade Induced Apoptosis in MDA-MB-231 Cells Activation of ERK 1/2

**Figure 4:**
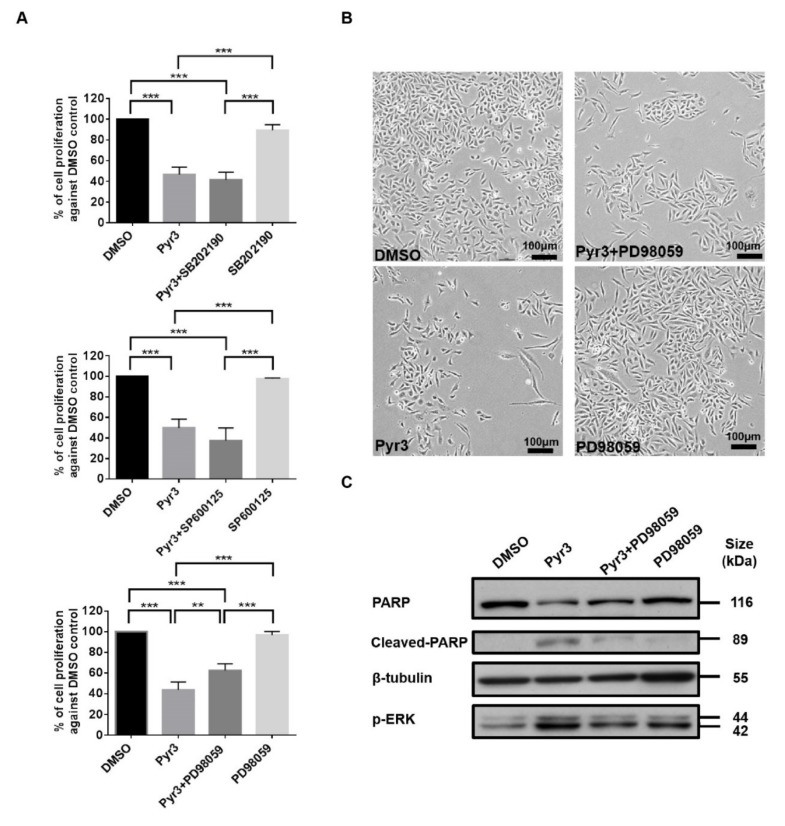
TRPC3 blockade induced apoptosis in MDA-MB-231 cells through activation of ERK 1/2. (**A**) decrease in the percentage of cell proliferation in response to Pyr3 (1.0 μM for 72 h) was attenuated by pre-treatment with ERK1/2 inhibitor PD98059 (5.0 μM for 24 h) as measured by MTT assay. Pre-treatment of MDA-MB-231 cells with p38 MAPK inhibitor SB202190 (1.0 μM for 24 h) and JNK inhibitor SP600125 (1.0 μM for 24 h) did not reverse the effect of Pyr3. Values are mean ± SEM (*n* = 3). ** *p* < 0.01 and *** *p* < 0.001; (**B**) cell density and cell morphology of the four treatment groups (DMSO only, DMSO followed by Pyr3, PD98059 followed by Pyr3 and PD98059 only) were observed under phase-contrast microscope. Scale bar: 100 μm; (**C**) representative Western blots showing that increased level of cleaved PARP and phosphorylated ERK1/2 proteins induced by Pyr3 was attenuated by pre-treatment with ERK1/2 inhibitor PD98059 (5.0 μM for 24 h).

To further elucidate the signaling cascade leading to apoptosis in MDA-MB-231 as induced by TRPC3 blockade, we studied whether p38 MAPK, ERK 1/2 and/or JNK were involved by co-application of MAPK inhibitors [18] with Pyr3. While pre-treatment with p38 MAPK inhibitor SB202190 (1.0 μM for 24 h) or JNK inhibitor SP600125 (1.0 μM for 24 h) did not reverse the effect of Pyr3 (1.0 μM for 72 h) on cell viability, the decrease of cell proliferation by Pyr3 was attenuated by MEK-ERK inhibitor PD98059 (5.0 μM for 24 h) (Figure 4A). Consistently, cell density of the group treated with PD98059 followed by Pyr3 was relatively higher than that of the group treated with DMSO followed by Pyr3 as observed under the phase-contrast microscopy (Figure 4B). Western blot showed that PARP cleavage and phosphorylation of ERK 1/2 induced by Pyr3 was attenuated by PD98059 treatment (Figure 4C). These results suggested that TRPC3 blockade induces apoptosis in MDA-MB-231 cells through activation of ERK 1/2.

### 2.5. Involvement of RASA4 in TRPC3-Mediated Calcium Signaling Transduction

**Figure 5:**
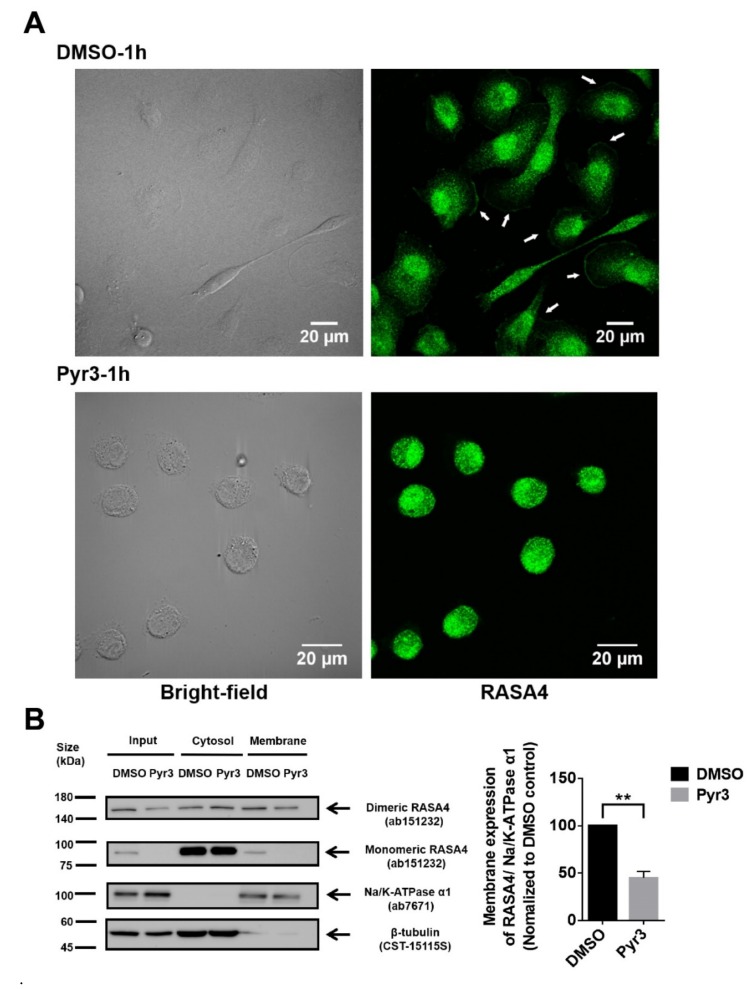
Blocking TRPC3 in MDA-MB-231 downregulated RASA4 expression on the plasma membrane. (**A**) representative confocal images showing the subcellular distribution of RASA4 (green) in MDA-MB-231. Cells were fixed after DMSO/ Pyr3 treatment for 1 h. Merging fluorescence image with bright field image suggested that RASA4 expression on the plasma membrane (indicated by white arrows) was decreased in most of the Pyr3-treated cells. Scale bar: 20 μm; (**B**) subcellular fractionation followed by Western blot analysis confirmed that blocking TRPC3 by Pyr3 (1.0 μM for 1 h) significantly decreased the expression of RASA4 proteins in the membrane fraction of MDA-MB-231 when compared to DMSO control group. Na/K-ATPase α1 was used as a membrane protein marker and β-tubulin was used as a cytosolic protein marker. Band density (total RASA4 normalized to Na/K-ATPase α1) was calculated by Image J software. Values are mean ± SEM (*n* = 3). ** *p* < 0.01.

To elucidate the role of TRPC3 in regulating calcium signaling transduction, expression of RASA4 in MDA-MB-231 was explored. RASA4 is a Ca^2+^-dependent Ras-MAPK pathway suppressor. RASA4 is able to translocate onto the cell surface plasma membrane in response to an increased cytosolic calcium, where it converts Ras from the active GTP-bound state to the inactive GDP-bound state, and therefore no longer activates downstream pathways [19,20]. Immunocytochemical staining followed by confocal microscopy showed that RASA4 was located on the plasma membrane of the MDA-MB-231 (Figure 5A). In addition, previous studies showed that RASA4 can exist as monomer or dimer [21]; we found that both monomeric and dimeric RASA4 were present in MDA-MB-231 cells (Figure 5B). Interestingly, RASA4 expression on the plasma membrane was consistently decreased in most of the Pyr3-treated MDA-MB-231 cells (Figure 5A; also see Figure S4 for the heterogeneous changes of cellular morphologies in response to Pyr3). Subcellular fractionation followed by Western blot analysis confirmed that expression of both monomeric and dimeric RASA4 were downregulated in the plasma membrane fraction of Pyr3-treated cells when compared to DMSO-treated cells (Figure 5B). These results suggested that RASA4 is downstream of the calcium influx mediated by TRPC3.

## 3. Discussion

**Figure 6:**
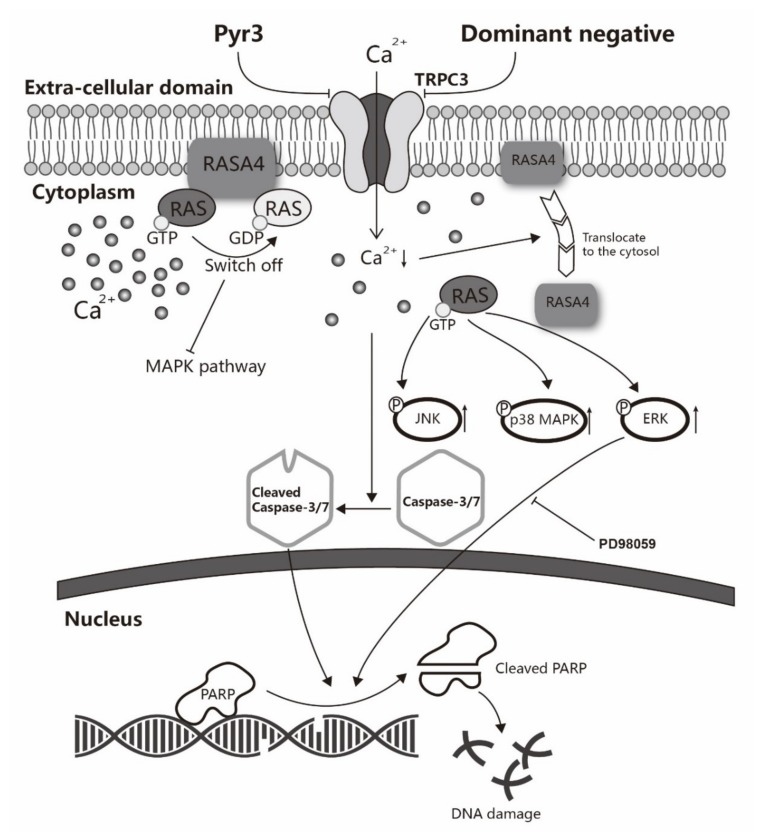
A schematic diagram explaining how TRPC3 acts as an anti-apoptotic regulator via RASA4-MAPK pathway in MDA-MB-231. TNBC cell line MDA-MB-231 overexpresses TRPC3 on the plasma membrane. Functional presence of TRPC3 regulates calcium entry across the plasma membrane into the cytosol. TRPC3 is oncogenic in MDA-MB-231 with suppression of ERK1/2 phosphorylation. TRPC3 blocker Pyr3 and DN of TRPC3 inhibit cell proliferation and induce apoptosis in a caspase-dependent manner. Blocking TRPC3 activates MAPK pathways in MDA-MB-231. RASA4, a Ca^2+^-promoted Ras-MAPK pathway suppressor, is located on the plasma membrane of MDA-MB-231 where it inhibits Ras-MAPK pathway. Ca^2+^ influx through TRPC3 channel sustains the expression of RASA4 on the cell plasma membrane. Blocking TRPC3 decreases the cytosolic Ca^2+^ level; this, in turn, decreases the amount of RASA4 on the plasma membrane, with concomitant activation of MAPK pathway. Taken together, functional TRPC3 channels over-expressed on the plasma membrane contribute to the apoptosis resistance of MDA-MB-231 cells through regulating Ca^2+^-dependent signaling cascade. Our study suggests that TRPC3 can be exploited as a potential molecular-based therapeutic target for TNBC.

The major novel findings of this study are as follows: (1) TNBC cell line MDA-MB-231 over-expresses TRPC3 channel on the plasma membrane; (2) functional presence of TRPC3 regulates extracellular calcium entry across plasma membrane into cytosol; (3) blockade of TRPC3 decreases MDA-MB-231 proliferation but does not affect cell cycle distribution; (4) blockade of TRPC3 induces apoptosis via the activation of ERK1/2 in MDA-MB-231; (5) RASA4, a Ca^2+^-promoted Ras-MAPK pathway suppressor, is located on the plasma membrane of MDA-MB-231; blockade of TRPC3 causes the translocation of RASA4 from the plasma membrane to the cytosol. Taken all these findings together, we highlight a key functional role of the TRPC3-RASA4-MAPK signaling cascade in maintaining the proliferation and apoptosis resistance of TNBC cells. A schematic illustration is shown in Figure 6.

Over-expressed TRPC6 was found to promote breast cancer cell growth and metastasis [22]. TRPC1 was reported to play an important role in basal-like breast cancer cell migrations with regulation of the epithelial to mesenchymal transition (EMT) procedure [23]. TRPC5 was reported to be essential for the survival of adriamycin-resistant MCF-7 cells through induction of the expression of a key efflux transporter P-glycoprotein [24]. In our present study, we aimed to identify a potential molecular therapeutic target of TNBC cells distinguished from hormone receptor positive breast cancer cells. A previous study has reported the abnormal upregulation of TRPC3 and TRPC6 in breast cancer tissues from patients [11]; the differential expression of TRPC3 in MCF-7 and MDA-MB-231 has attracted our attention. In our current study, by Western blot and immunocytochemistry, TRPC3 was found to be over-expressed on the plasma membrane of MDA-MB-231 when compared to MCF-7, consistent with this previous study [11]. In yet other studies, TRPC3 was reported to contribute to the proliferation of ovarian cancer cells and lung cancer cells [25,26,27,28,29]; our current findings that the upregulated TRPC3 in MDA-MB-231 plays a positive role in cancer progression are in line with those previous studies. 

Expression of DNA repair genes are downregulated in TNBC; and this has been suggested to increase the effectiveness of DNA damage response inhibitors for the treatment of TNBC [30]. Patients with basal-like TNBC are suggested to be preferentially treated with agents that engage DNA damage signaling response pathways (e.g., PARP inhibitors) [1]. We found that blocking TRPC3 induced apoptosis of MDA-MB-231 which was characterized by morphological and biochemical changes including cell shrinkage, membrane blebbing, DNA fragmentation, cleavage of caspase-3/7 and PARP [31]. It has been known for long that caspases-3/7 cleaves PARP and inactivates its DNA-repairing abilities during apoptosis [32]. In our study, TRPC3 blockade was found to increase the amount of cleaved caspase-3/7, suggesting that blocking TRPC3 induces caspase-dependent apoptosis in MDA-MB-231. 

Our study revealed that TRPC3 was oncogenic in MDA-MB-231 with suppression of ERK1/2 phosphorylation. Dysregulation of Ras-MAPK pathway is commonly observed in cancer [33]. Multiple anti-cancer drugs targeting Ras-MAPK pathway are currently under clinical trials [34]. While MDA-MB-231 is a KRas mutant (G13D) cell line [35], we found that there was no significant change of cell proliferation in MEK-ERK inhibitor PD98059-treated MDA-MB-231 cells. In contrast, decrease of cell proliferation caused by TRPC3 blockade was attenuated in PD98059 pre-treated cells. Therefore, our results suggested that mutated KRas may lead to constitutive activation of other MAPK pathway targets but not ERK1/2. In fact, reversible phosphorylation and dephosphorylation of MAPK subfamilies at serine and threonine residues play dual roles in cell death [36]. On one hand, activation of MEK-ERK pathway was commonly regarded to serve an anti-apoptotic function by protecting caspase-9 from cleavage [37]. On the other hand, constitutively activated ERK was reported to sensitize cancer cells to different chemotheraputic agents [38,39]. Our results indicated that blocking TRPC3 caused apoptosis through activation of ERK1/2.

A loss of intracellular Ca^2+^ homeostasis triggers cell apoptosis [40,41]. Endoplasmic reticulum (ER)-Ca^2+^ store depletion and mitochondrial Ca^2+^ uptake are key pro-apoptotic players [8,26,42]. In addition, Ca^2+^ influx through TRP channels was reported to act as anti-apoptotic regulators through activation of Ca^2+^-sensitive downstream pathways [43,44,45]. Our present study firstly demonstrated that Ca^2+^-dependent mechanism for activation of MAPK pathway caused by TRPC3 blockade is mediated through the TRPC3-RASA4-MAPK signaling cascade. In RAS mutated tumor cells, the signaling transduction upstream of MAPK pathway is complex. Both RASAL2 and RASA4 are members of RAS GTPase-activating proteins (RAS-GAPs) catalyzing GTP into GDP and therefore inactivating RAS. RASAL2 has recently been found to activate RAC1 and contribute to TNBC tumorigenesis [46]. On the other hand, numerous previous studies showed that plasma membrane RASA4 switched off the MAPK pathway in response to the elevating intracellular Ca^2+^ [19,20,21]. In our study, RASA4 was found to be over-expressed on the plasma membrane of MDA-MB-231 when compared to MCF-7 where it inhibited MAPK pathway. RASA4 on MDA-MB-231 plasma membrane was decreased in response to a decreased free Ca^2+^ concentration induced by Pyr3, with concomitant activation of MAPK pathway.

In summary, our study found that in TNBC MDA-MB-231 cells, Ca^2+^ influx through TRPC3 channel sustains the presence of RASA4 on the plasma membrane where it inhibits Ras-MAPK pathway, leading to proliferation and apoptosis resistance. Our study reveals this novel TRPC3-RASA4-MAPK signaling cascade in TNBC cells and suggests that TRPC3 may be exploited as a potential therapeutic target for TNBC.

## 4. Materials and Methods

### 4.1. Cell Culture

This study was approved by the Ethics Committee (the Clinical Research Ethics Committee (CREC) code is 2014.236), the Chinese University of Hong Kong and followed the tenant of the Declaration of Helsinki. Two human breast cancer cell lines, MDA-MB-231 (ER–, PR– and HER2–) and MCF-7 (ER+, PR+/− and HER2–) (American Type Culture Collection, Manassas, VA, USA), were selected as our in vitro research model. They were cultured at 37 °C under an atmosphere of 5% CO_2_/95% air. MCF-7 cells were cultured in the phenol red-free RPMI 1640 medium (Invitrogen, Carlsbad, CA, USA) supplemented with 10% heat-inactivated fetal bovine serum (FBS) (Invitrogen), whereas MDA-MB-231 cells were cultured in the same RPMI 1640 medium supplemented with 5% heat-inactivated FBS.

### 4.2. Treatment Regimen

The effects of TRPC3 blocker Pyr3 (Tocris, Bristol, UK) on MDA-MB-231 were investigated. Cells were seeded at the density of 6.67 × 10^4^ MDA-MB-231 cells cm^−2^ on 0.1% gelatin (Sigma-Aldrich, Missouri, MO, USA)-coated dish (96-well dish for MTT and Trypan blue exclusion assay, 24-well dish for immunocytochemistry and 6-well dish for protein harvesting) and were allowed for adhesion overnight. MDA-MB-231 cells were then treated with TRPC3 blocker Pyr3 or DMSO (solvent control) for three to five days. SP600125 (JNK inhibitor, 1 μmol/L, Tocris), PD98059 (MEK-ERK inhibitor, 5 μmol/L, Tocris), and SB202190 (p38 MAPK inhibitor, 1 μmol/L, Tocris) were used to treat cells for 24 h prior to Pyr3 exposure. Trypan blue exclusion, MTT, cell cycle, Western blot and immunocytochemistry analyses were then performed.

### 4.3. Western Blot

MCF-7 and MDA-MB-231 cell lysates were prepared and Western blot was performed as previously described [47]. To assay for the presence of TRPC3, 1:1000 rabbit anti-TRPC3 (Alomone) and 1:1000 mouse anti-TRPC3 (Santa Cruz) were used. To validate the specificity of the anti-TRPC3 antibody, the anti-TRPC3 was pre-incubated with its blocking peptide according to the manufacturer’s instructions for 2 h at 37 °C prior to the membrane incubation. To assay for apoptotic cell death, primary antibodies 1:1000 rabbit anti-caspase-7, 1:200 rabbit anti-caspase-3, 1:1000 rabbit anti-PARP (Cell Signaling, Danvers, MA, USA) were used. To assay for MAPK pathway involvement, 1:1000 rabbit anti-phosphorylated or total p38 MAPK, ERK1/2 and JNK (Cell Signaling, Danvers, MA, USA) were used. In all cases, the membranes were stripped and probed with 1:1000 rabbit anti-β-tubulin (Cell Signaling) as an internal control. After primary antibody probing, membranes were washed in TBST, and incubated with HRP-conjugated secondary antibody (Dako, Glostrup, Denmark) in the dilution of 1:3000 for 1 h at room temperature. Protein expression was detected by enhanced chemiluminescent substrate (Pierce, Thermo Fisher Scientific, Waltham, MA, USA) and protein bands were visualized by film exposure. The density of the bands was quantified using Image J software (version 1.48v, National Institutes of Health, Bethesda, MD, USA).

### 4.4. Immunocytochemistry

MCF-7 and MDA-MB-231 cells were seeded on 0.1% gelatin-coated glass coverslips in 24-well culture plates (Thermo Fisher Scientific) for 24 h and were allowed to proliferate for 48 h. Cells were then fixed with 2% paraformaldehyde (Sigma-Aldrich) for 10 min at 37 °C, then rinsed in PBS twice for 5 min, and subsequently incubated in 0.1% Triton X-100 (Sigma-Aldrich) for 15 min. Coverslips were then washed with PBS twice, and incubated in a blocking solution containing 2% BSA and 5% normal goat serum (NGS) (Invitrogen) for 1 h followed by an overnight incubation in the blocking solution containing antibodies at 4 °C in the dark. To assay for the presence of TRPC3, the coverslips were incubated with 1:100 rabbit anti-TRPC3 (Abcam) and 1:100 mouse anti-TRPC3 (Abnova), respectively. To assay for the presence of RASA4, 1:100 rabbit anti-RASA4 (Abcam) was used. After three times being washed with PBS supplemented with 0.1% Tween (Sigma-Aldrich), secondary antibodies, 1:100 Alexa Fluor 488/594 goat anti-mouse/rabbit (Invitrogen), were diluted in 1% NGS/PBS and applied to incubate the cover slides for 1 h at room temperature. Then 1:5000 DAPI (Roche, Basel, Switzerland) in PBS was used to stain nuclei for 10 min at room temperature. Slides were affixed with mounting medium (Dako, Carpinteria, CA, USA) and viewed using an Olympus FluoView FV1000 confocal laser scanning microscope with a 60 × objective. Images were analyzed using the FV1000 software (Olympus, Tokyo, Japan).

### 4.5. Subcellular Fractionation Followed by Western Blot

Whole cell pellets of MDA-MB-231 were fractionated into cytosol and membrane fractions. Cells were lysed by hypotonic fractionation buffer (0.32 M sucrose, 5 mM Tris at pH 7.4) freshly supplemented with protease inhibitor cocktail (Roche). After vortex and passing through a syringe with a 27 gauge needle for ten times, the supernatant (membrane and cytosol) and pellet (nuclear fraction) were separated by centrifugation at 500× *g* for 10 min at 4 °C. The supernatant was further centrifuged at 100,000× *g* for 1 h at 4 °C to separate the cytosol and the membrane fraction. The pellet was resuspended with membrane resuspension buffer (20 mM HEPES, 1 mM EDTA, 10% glycerol, 120 mM KCl and 2% Triton X-100) freshly supplemented with protease inhibitor cocktail. Protein concentration of each fraction was determined using the Bradford assay (Bio-Rad, Hercules, CA, USA). α1 sodium/potassium-ATPase (Na/K-ATPase α1) and β-tubulin were used as the protein makers of the membrane fraction and cytosolic fraction, respectively. Mouse anti-Na/K-ATPase α1 (1:1000, Abcam) and rabbit anti-β-tubulin (1:1000, Cell Signaling) were used in primary antibody incubation step and all the subsequent processes for Western blot were conducted as described above under ‘4.3 Western Blot’.

### 4.6. Confocal Ca^2+^ Imaging

Confocal Ca^2+^ imaging using Fluo-4 AM (Thermo Fisher Scientific) was performed as previously described [17]. Drugs including adenosine-triphosphate disodium salt hydrate (ATP) (Sigma), CaCl_2_ (Sigma) and Pyr3 were added at their appropriate concentrations at a given time. Equal volumes of dimethyl sulfoxide (DMSO) (Sigma) were also added in the solvent control group. Raw traces reflected the changes in cytosolic Ca^2+^ level were expressed as F/F0 which was defined by the fluorescence intensity at a given time normalized to its baseline. Data was analyzed using with FV1000 software (Olympus).

### 4.7. Proliferation Assay

MDA-MB-231 cells were treated with TRPC3 blocker Pyr3 or DMSO for 3–5 days. Previous studies have shown that expression of the N-terminal fragment of TRPC3 (N-terminal domain consisted of amino acids 1–302 of human TRPC3) would lead to a dominant negative (DN) effect on TRPC3 channel function [17,48]. Recombinant adenoviruses transferring green fluorescent protein (GFP) and DN of TRPC3 were constructed previously by our group [17] and were used to infect MDA-MB-231 cells. Cell viability and cell proliferation were measured by MTT assay. Viable cell numbers were measured by Trypan blue exclusion assay as previously described [47].

### 4.8. Propidium Iodide (PI) Staining Followed by Flow Cytometry for Cell Cycle Analysis

Cells were seeded at the density of 3.33 × 10^4^ MDA-MB-231 cells cm^−2^ on the 100-mm cell culture dishes (Cellstar, Greiner bio-one, Kre Austria). In addition, 1 × 10^6^ cells per treatment group were harvested with 0.05% trypsin-EDTA (Invitrogen), then fixed with 70% ethanol (Sigma) on ice for 30 min. Cells were then centrifuged at 200× *g* and the cell pellet was resuspended with staining solution containing 2 μg/mL PI (Sigma) and 10 mg/mL RNase A (Thermo Fisher Scientific) in PBS for 30 min in dark at 37 °C and analyzed using a BD FACSVerse flow cytometer (BD Biosciences, San Jose, CA, USA). The percentages of viable cells residing in G0/G1, S, and G2/M phases and apoptotic cells residing in sub-G1 phase were calculated using the ModFit LT software (Verity Software House, Topsham, ME, USA).

### 4.9. Fluorescence Imaging

Living cell morphology of MDA-MB-231 cells and green fluorescence of adenovirus-infected MDA-MB-231 cells were captured using Nikon TE300 eclipse microscope with a 10× objective. For observation of programmed cell death, living cells were stained with 100 nM Mitotracker Red CMXRos (Invitrogen) at 37 °C under an atmosphere of 5% CO_2_/95% air for 30 min. Cells were then fixed with 2% paraformaldehyde (Sigma-Aldrich) for 10 min at 37 °C, rinsed in PBS twice for 5 min, and subsequently incubated in 0.1% Triton X-100 (Sigma-Aldrich) for 15 min. 1:5000 DAPI (Roche) in PBS was used to stain nuclei for 10 min at room temperature. Cell morphology was observed using the Olympus FluoView FV1000 confocal laser scanning microscope with a 60× objective and further analyzed using the FV1000 software [49].

### 4.10. Statistical Analysis

Each experiment was repeated at least three times. The results were expressed as mean ± SEM. Statistical significance between two groups of means was determined by student’s *t*-test. Statistical significance between three or more groups of means was determined by analysis of variance (ANOVA). *p <* 0.05 was considered to be statistically significant.

## 5. Conclusions

In conclusion, we demonstrated that Ca^2+^ influx through TRPC3 channels sustained the localization of RASA4 on the plasma membrane, suppressed the MAPK pathway and protected MDA-MB-231 cells from apoptotic cell death. A blockade of TRPC3 induced apoptosis and sensitized MDA-MB-231 cells to chemotherapeutic agents. The present study provides a rational account that functional TRPC3 over-expressed on the plasma membrane of MDA-MB-231 cells can be exploited as a potential molecular-based therapeutic target for TNBC [50].

## Supplementary Materials

The following are available online at https://www.mdpi.com/2072-6694/11/4/558/s1, Figure S1: The expression of TRPC3 in MCF-7 and MDA-MB-231, Figure S2: Confocal images showing typical apoptotic morphological changes in MDA-MB-231 cells, Figure S3: Levels of cleaved caspase-3 were increased due to TRPC3 blockade, Figure S4: Heterogeneous changes in cell morphology in response to Pyr3.
