# Supplementary material for: TRPC3 Regulates the Proliferation and Apoptosis Resistance of Triple Negative Breast Cancer Cells through the TRPC3/RASA4/MAPK Pathway
Source: Cancers (Basel). 2019 Apr 18;11(4):558. doi: 10.3390/cancers11040558 (PMC6520729; doi:10.3390/cancers11040558)
Supplement: Supplementary file 1 [file cancers-11-00558-s001.pdf]

Supplementary Materials: TRPC3 Regulates the Proliferation and Apoptosis Resistance of Triple Negative Breast Cancer Cells through TRPC3/RASA4/MAPK Pathway

Yan Wang, Yan-Xiang Qi, Zenghua Qi and Suk-Ying Tsang

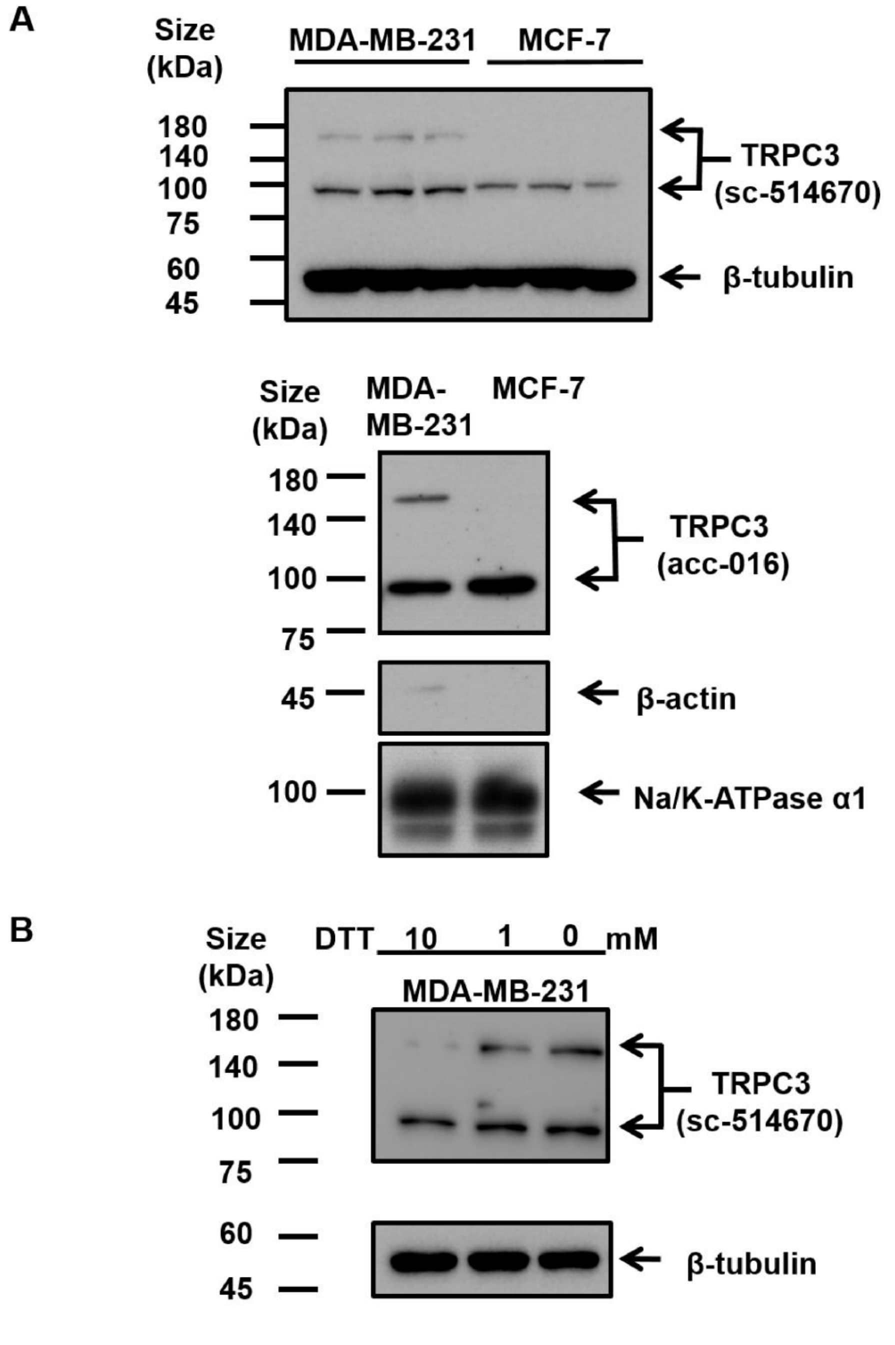

Figure S1. The expression of TRPC3 in MCF-7 and MDA-MB-231. (A) (Upper panel) Representative Western blots showing the expression of TRPC3 in different batches of MCF-7 and MDA-MB-231

protein lysates. β-tubulin was used as an internal control. (Lower panel) Subcellular fractionation followed by Western blot analysis confirmed that the over-expressed TRPC3 protein represented by the band between 140 and 180 kDa was enriched in the membrane fraction of MDA-MB-231 but not in that of MCF-7. Na/K-ATPase $\alpha 1$ was used as a membrane protein marker and β-actin was used as a cytosolic protein marker. (B) A form of TRPC3 was found to be sensitive to the reducing agent dithiothreitol (DTT) in MDA-MB-231. The TRPC3 band between 140 and 180 kDa (which was upregulated in MDA-MB-231) became faded after treatment of the sample with 1mM and 10 mM DTT at 95°C for 10 minutes.

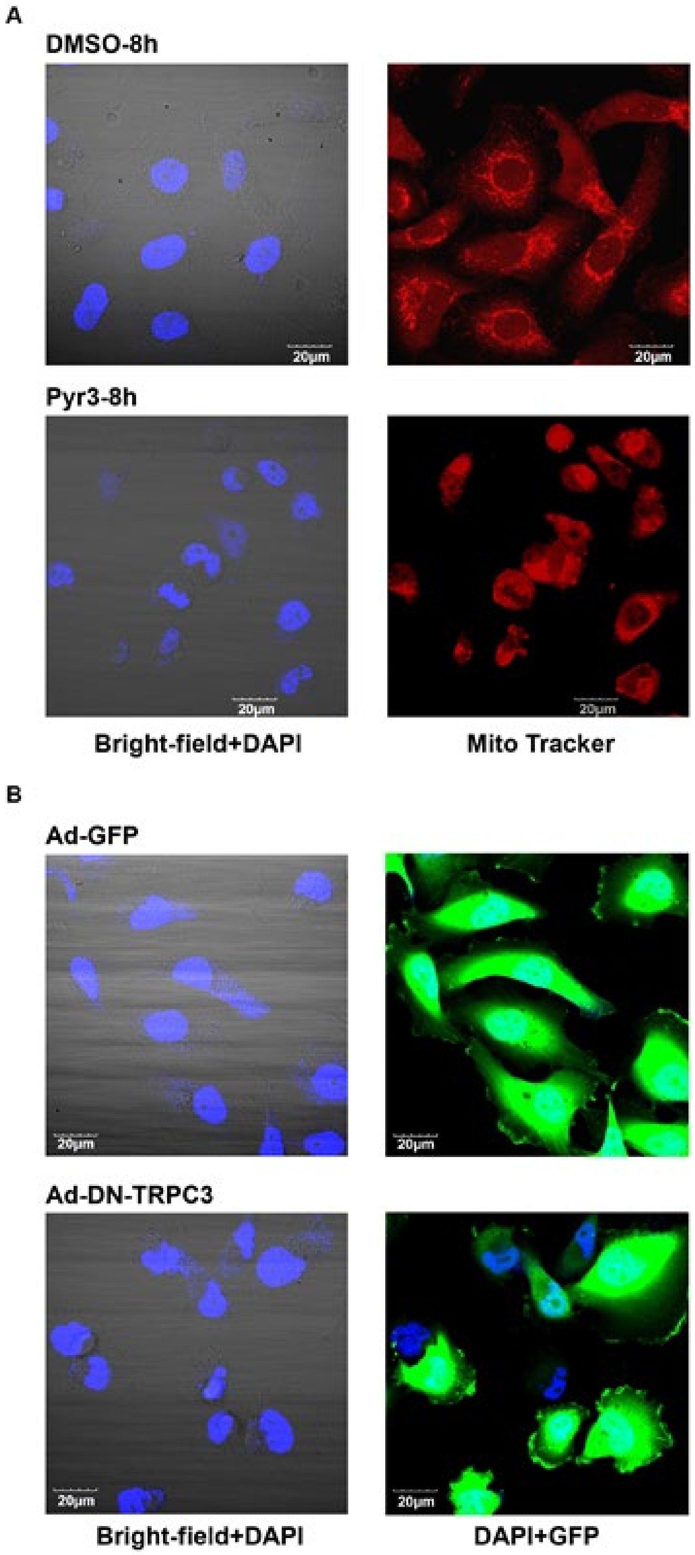

Figure S2. Confocal images showing typical apoptotic morphological changes in MDA-MB-231 cells. (A) A series of typical apoptotic morphologies were observed in the Pyr3-treated cells including cell shrinkage, membrane blebbing, mitochondrial fragmentation and nuclear condensation. Cells were fixed after DMSO/ Pyr3 treatment for 8 h. Mitochondria were stained by MitoTracker® Red CMXRos (red) and nuclei were stained by DAPI (blue). (B) Adenovirus (Ad) harboring GFP (Ad-GFP) or DN of TRPC3 (Ad-DN-TRPC3) were used to infect MDA-MB-231 for 48 h Cells were fixed at 24 h after adenoviruses withdrawal. Cell shrinkage and nuclear condensation were observed in the Ad-DN-TRPC3 infected cells. Scale bars: 20 μm.

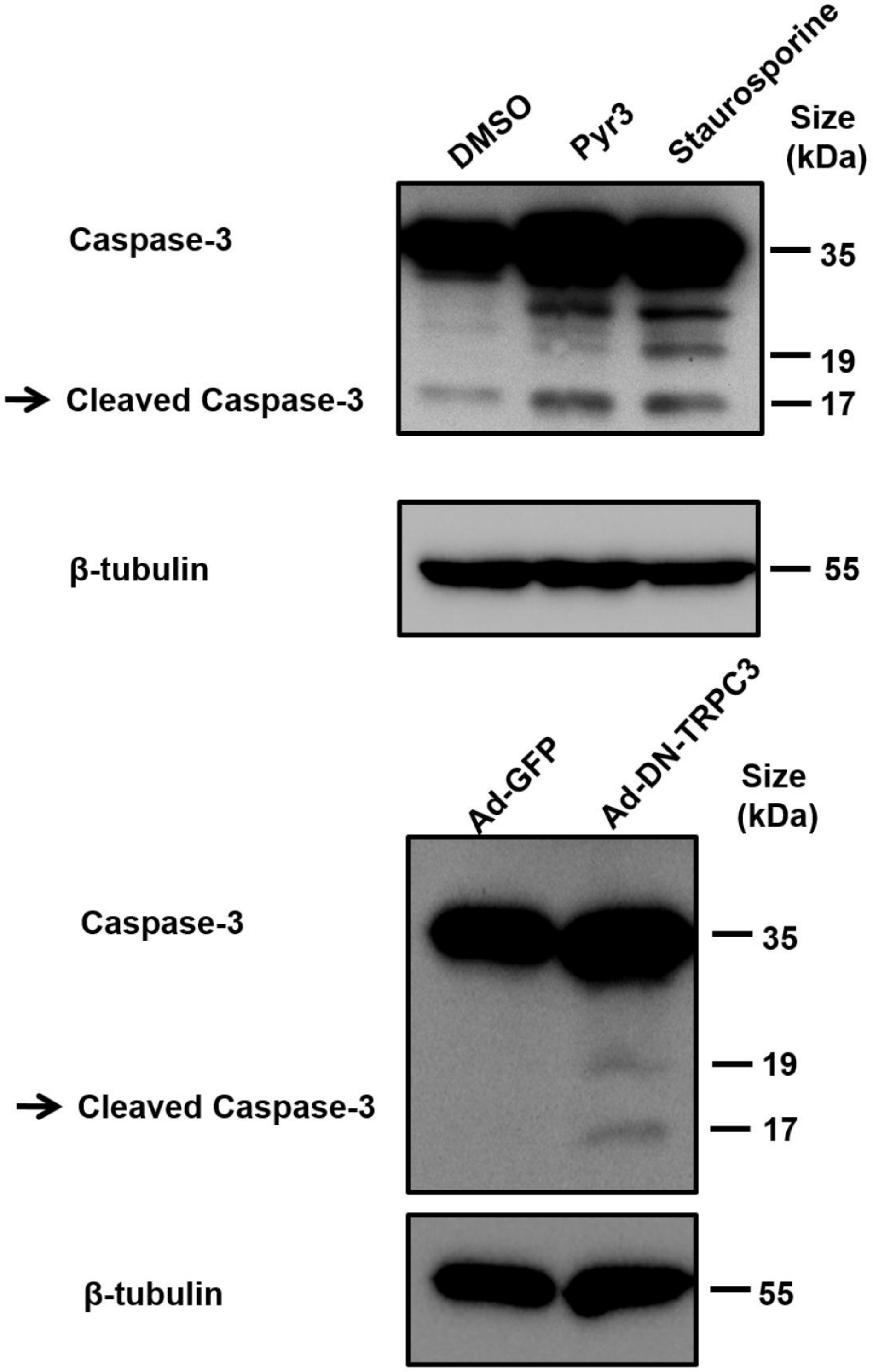

Figure S3. Levels of cleaved caspase-3 were increased due to TRPC3 blockade. (Upper panel) Representative Western blots showing that levels of cleaved caspase-3 were increased in Pyr3-treated MDA-MB-231 cells when compared to DMSO control group. MDA-MB-231 cells treated with 0.1 μM staurosporine (apoptosis inducer) for 24 h was used as positive control for detection of bands of cleaved caspase-3 proteins. β-tubulin was used as an internal control. (Lower panel) Cleaved caspase-3 was also detected in Ad-DN-TRPC3-infected MDA-MB-231 cells. TRPC3 blockade induced apoptosis of MDA-MB-231 in a caspase-dependent manner.

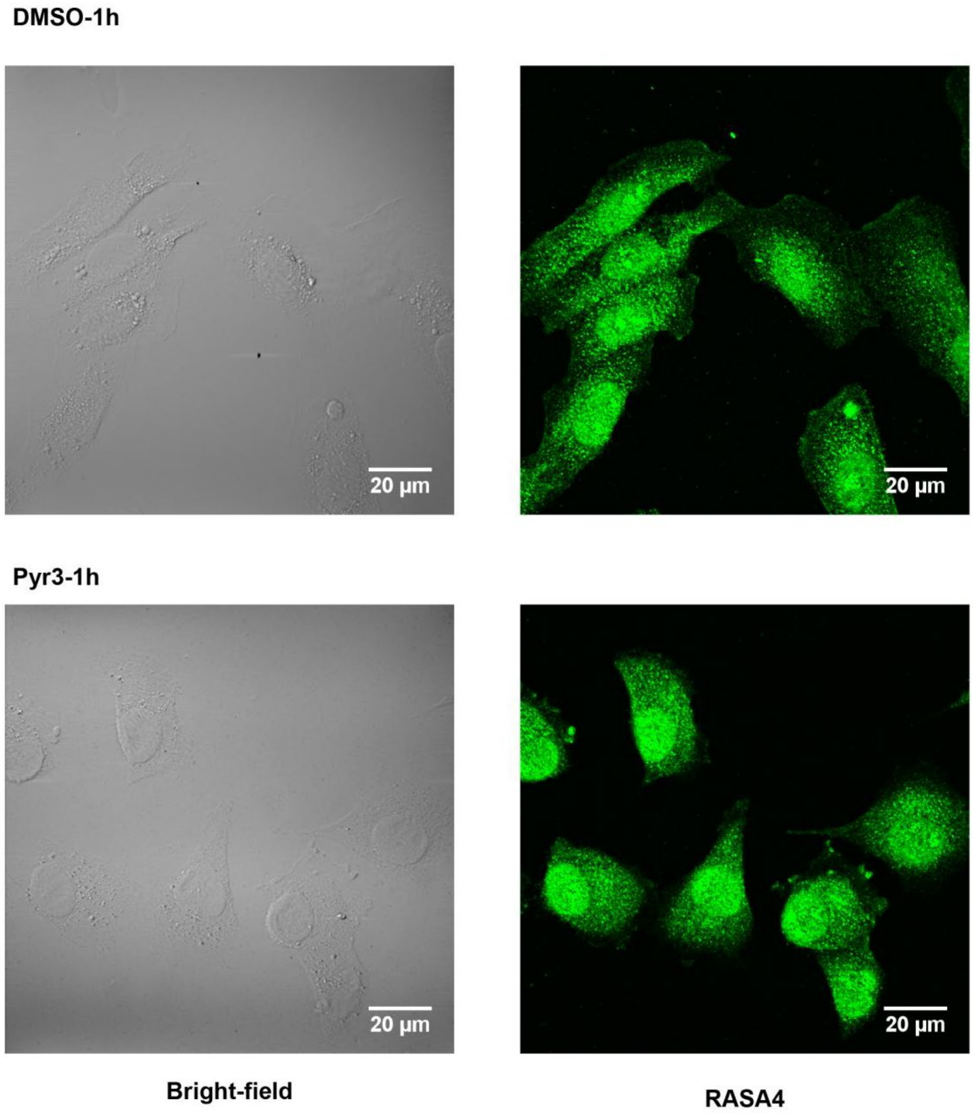

Figure S4. Heterogeneous changes in cell morphology in response to Pyr3. Blocking TRPC3 consistently decreased RASA4 expression on the plasma membrane of MDA-MB-231 cells but different cell morphology could be observed (c.f. Figure 5A).
